# The Impact of a Leadership Support Programme on Care Home Residents and Their Families: A Qualitative Study From the Perspective of Participating Care Home Leaders

**DOI:** 10.1111/opn.70054

**Published:** 2025-11-16

**Authors:** Brighide Lynch, Assumpta Ryan, Sonja McIlfatrick, Sarah Penney, Rosemary Bradley, Marie O'Neill, Claire McCauley, Esther‐Ruth Beck, Anthony Curran, Una Hume, Deborah Muldrew, Paul Slater

**Affiliations:** ^1^ School of Nursing and Paramedic Science and Institute of Nursing and Health Research Ulster University Londonderry UK

**Keywords:** care homes, care of older people, leadership, leadership development, relationship‐centred care, relatives, residents

## Abstract

**Background:**

Older people are entering care homes with more complex conditions and higher levels of physical and cognitive impairment than in previous years. Internationally, it is recognised that there is a need for high‐performing leaders who can inspire and support colleagues to use their initiative and respond to the needs of an increasingly frail population in long‐term care. The My Home Life Leadership Support Programme is specifically designed to meet the unique needs of care home managers and other staff with leadership roles in their care homes. The programme is grounded in relational, appreciative and collaborative approaches to creating a positive culture of mutually respectful relationships. These relationships include those that are between individuals who use services, their families and care home staff, and between care homes and the wider community.

**Aim:**

To explore participating care home leaders' perspectives of the impact of the My Home Life Leadership Support Programme on people who live in care homes and their families.

**Methods:**

A qualitative descriptive approach drawing on two different data sources was used in the study. Qualitative summative data were collected at the end of the programme using one‐to‐one semi‐structured interviews with participants (*n* = 56), and detailed field notes were captured by the My Home Life facilitators over the course of the programme. Thematic analysis was used to analyse interview data. Conventional content analysis was used to analyse the facilitators' documentary evidence. The two analytical techniques were combined for the final results using the process of cognitive mapping.

**Results:**

Three key themes were identified by care home leaders as mattering most to residents and their families. These were (1) supporting residents and families to share their feelings; (2) enhancing relationships between residents, relatives and staff; and (3) involving residents and families in decision‐making.

**Conclusions:**

The study provides significant evidence of the impact of the My Home Life Leadership Programme on care home leaders' ability to enhance the care experience of residents and relatives and create a relationship‐centred culture.

**Implications for Practice:**

My Home Life resources and approaches can support care home leaders in facilitating caring conversations that build trust and engagement, and enhance relationships between residents, relatives and staff.

**Summary**

- What does this research add to existing knowledge in gerontology?
  ○ This paper contributes to a gap in knowledge about care home leaders' perceptions of the impact of leadership on people who live in care homes and their families.
  ○ Care home leaders' perceptions of using My Home Life inquiry tools to facilitate conversations are that they can support residents and relatives in care homes to articulate meaning and share the complex range of emotions they are feeling.
- What are the implications of this new knowledge for nursing care for and with older adults?
  ○ Care home leaders have a crucial role in developing relationships, supporting residents, relatives and staff to appreciate their own part in promoting voice, choice and control in care homes.
  ○ The consistent modelling of appreciative inquiry and caring conversations by care home leaders is highlighted as being helpful in building trust and enhancing relationships between residents, relatives and staff.
- How could the findings be used to influence practice, education, research, and policy?
  ○ The findings have significant applicability across all long‐term care settings, nationally and internationally where staff work collaboratively with service users and families to create a relationship‐centred culture.
  ○ Future research on the impact of leadership in care homes should include the perspective of care home residents and relatives in order to add to the literature on what matters most about life in a care home.

## 1 Introduction

The global population of people aged 65 years or over increased from 6% in 1990 to 9% in 2019. This proportion is projected to rise further to 16% by 2050, resulting in one in six people in the world aged 65 years or older (United Nations Report 2019). In 2019, across Organisation for Economic Co‐operation and Development (OECD) countries, an average of 10.7% of people aged 65 and over received long‐term care, either at home or in long‐term care facilities. More recent figures show an average of 46 beds per 1000 people aged 65 and over in 2021 with the vast majority of these beds located in long‐term care facilities (OECD 2023). In the United Kingdom, an estimated 426,000 older people live in approximately 18,000 care homes (Age UK 2017). On 1 October 2018, the number of beds in all Registered Nursing and residential care homes across Northern Ireland had risen to 16,007 over a 10‐year period. According to the Regulation and Quality Improvement Authority (RQIA), this represented a 4% increase in the total number of beds in the sector (RQIA 2020). Older people are entering care homes with more complex conditions and higher levels of physical and cognitive impairment than in previous years. Burton et al. (2020) reported that, during the recent COVID‐19 pandemic, approximately one in six residents in care homes had confirmed COVID‐19. The combination of these complex health needs, together with the experience of transitioning to the care home, presents significant challenges for this most vulnerable group of people and their relatives (Brett 2023; O'Neill et al. 2020b). There is a growing recognition internationally of the need for high‐performing leaders who can inspire and support colleagues to use their initiative and respond to the needs of an increasingly frail population in long‐term care (Backman et al. 2021; Jeon et al. 2015; Siegel et al. 2012).

It is often difficult for care home managers to access opportunities for ongoing professional development and programmes that focus on leading change within their care home (Kelly and Kennedy 2017). Consequently, the need for a bespoke programme of leadership support and culture change has been widely advocated in the care home literature, nationally and internationally (Jeon et al. 2015; O'Neill et al. 2020a; Penney and Ryan 2018b). To date, there are few robust studies that explore effective leadership in care homes even though these settings provide care to some of the most vulnerable people in society (Jeon et al. 2015; Kennedy 2014). Based on the existing international research, several reviews recommend the need for a change in the culture of care within care homes to one that is more relationship‐centred led by a transformational leader (DoH 2015; Kelly and Kennedy 2017; NHS 2019; Zonneveld et al. 2021).

There are several leadership programmes rolled out across the globe that focus on developing the clinical, individual and organisational leadership capabilities of managers and leaders in healthcare settings. Such programmes include the Clinical Leadership in Aged Care Programme (CLiAC; Jeon et al. 2013) that originated in Australia; the LEADS in a Caring Environment (Dickson and Tholl 2014) developed in Canada; Vogelsmeier et al. (2010) developed the Leadership Development Academy for Registered Nurses in Long‐Term Care in Columbia; in Sweden, the National Board of Health and Welfare (NBHW) developed a leadership programme with the purpose of improving leadership skills among nursing home managers (Backman et al. 2021); the clinical leadership programme LEAP (learn, empower, achieve, produce) is an American programme designed specifically for aged‐care settings to enable job satisfaction and staff empowerment (Enghiad et al. 2022); and in the United Kingdom, the Royal College of Nursing Clinical Leadership Programme (RCN 2022) is a programme developed for clinical leaders in health and social care. While the majority of these leadership programmes are very strong on quantitative findings, they report very little on qualitative findings and most do not report on the impact of the leadership on staff and those receiving care (i.e., residents and families). Other leadership programmes exist but have been criticised for a tendency to focus on generic issues which are not always relevant to the unique needs and experiences of care home managers and leaders (Poels et al. 2020). The expected future shortage of managers and leaders in care services highlights the need for care homes to identify and develop their ‘rising stars’ to act as future leaders (Chenoweth et al. 2010). The My Home Life (MHL) Leadership Support Programme for care home leaders (Dewar et al. 2019; Owen and Meyer 2012; Penney and Ryan 2018a) is an innovative approach to leadership development with an emphasis on the importance of relationships with those who live, work and visit care homes. The MHL Leadership Support Programme is grounded in appreciation of the crucial role care home leaders play in shaping a positive culture of mutually respectful relationships. These relationships include those that are between residents, their families and care home staff, and between care homes and the wider community. This study reports on the impact of the programme on people who live in care homes and their families as perceived by participating care home leaders. Other papers will focus on the impact of the programme on staff and on the leaders themselves.

## 2 Materials and Methods

### 2.1 Aims and Objectives

The overall aim of this study was to explore participants' perspectives of the impact of the MHL Leadership Support Programme on people who live in care homes and their families and to make recommendations for policy and practice.

### 2.2 The MHL Leadership Support Programme (Intervention)

The MHL Leadership Support Programme (referred to as the ‘programme’ hereafter) was originally founded in 2006 by Help the Aged (now Age UK) in partnership with the National Care Forum and City University of London, and a core group of influential care organisations (www.myhomelife.org.uk). The programme is designed to meet the unique needs of care home managers by supporting them to improve quality of life for residents, relatives and staff. In addition to care home managers, the programme also targets deputy managers, ‘rising stars’ and other staff with leadership roles in their care homes (collectively referred to as ‘leaders’ hereafter). The programme began in England as a small project in 2006 and following collaboration with other partners, the initiative quickly spread across the United Kingdom to Wales in 2008, Scotland in 2012, and Northern Ireland in 2013. It is now recognised as an international initiative following its implementation in Australia in 2016 and Germany in 2017 (Penney et al. 2023). Over the years, some care homes have self‐funded the programme. However, in the main, the programme is funded by either local council adult social care teams, health authorities/departments or NHS Trusts. Participants are care home leaders who volunteer to participate in the programme and apply through expressions of interest. The programme is typically delivered to cohorts comprising 16–20 participants over 8–10 months and consists of full‐day workshops (*n* = 4) followed by monthly half‐day action learning sets (*n* = 7). Over the course of the programme, professional facilitators guide and support participants to advance their leadership skills, engage with the MHL conceptual frameworks and resolve the complex everyday issues that impact quality of life in care homes. An overview of the content of the workshops is presented in Appendix S1.

At the outset, MHL worked with over 60 academic researchers from universities across the United Kingdom to develop an evidence base for quality of life in care homes (NCHR&D Forum 2007). The review of evidence explored ‘what residents want from care homes’ and ‘what practices work in care homes’ and was subsequently updated 9 years later (MHL 2016). The eight themes emanating from the reviews have informed the content of the programme along with the evidence base underpinning relationship‐centred care (Nolan 2006) and caring conversations (Dewar et al. 2017). These three theoretical frameworks are presented in Table 1.

**TABLE 1:** The theoretical frameworks underpinning the MHL Leadership Support Programme.

Theoretical framework	Description
The Eight Best Practice Themes (NCHR&D Forum 2007; MHL 2016)	These are based on a literature review undertaken by over 60 academic researchers from universities across the UK which focused specifically on identifying: *‘What do residents want?*’ and ‘*What works well in care homes?’*. This study was subsequently updated in 2016. The evidence identified eight best practice themes, which were translated into a conceptual framework for use by the care home sector to support its practice. The eight themes are: Facilitating transitions; Maintaining Identity; Creating Community; Sharing Decision‐making; Improving Health & Healthcare; Supporting Good End of Life; Developing the workforce; and Promoting a Positive Culture
The Senses Framework (Nolan 2006)	The Senses Framework focuses on relationships and supports participants to achieve positive relationships between people living, working and visiting care homes, and between care services and the wider community. It suggests the need to consider what gives each individual a sense of security (to feel safe), belonging (to feel part of things), continuity (to experience links & connections), purpose (to have goals to aspire to), achievement (to make progress towards these goals) and significance (to feel that you matter as a person).
Caring Conversations (Dewar et al. 2017)	Caring Conversations support participants to deliver compassionate and relationship‐centred care using the 7 C's of Caring Conversations to: *celebrate* what is working well, *consider* the perspectives of others, *connect emotionally*, be *curious* and suspend judgement, be *courageous* and take positive risks, *collaborate* to make things happen and *compromis*e to focus on what is real and possible. The framework also helps to encourage and sustain genuine curiosity for ourselves and others, deepen inquiry, explore values, articulate tacit knowledge and acknowledge and express emotion without dispute or judgement.

**FIGURE 1:**
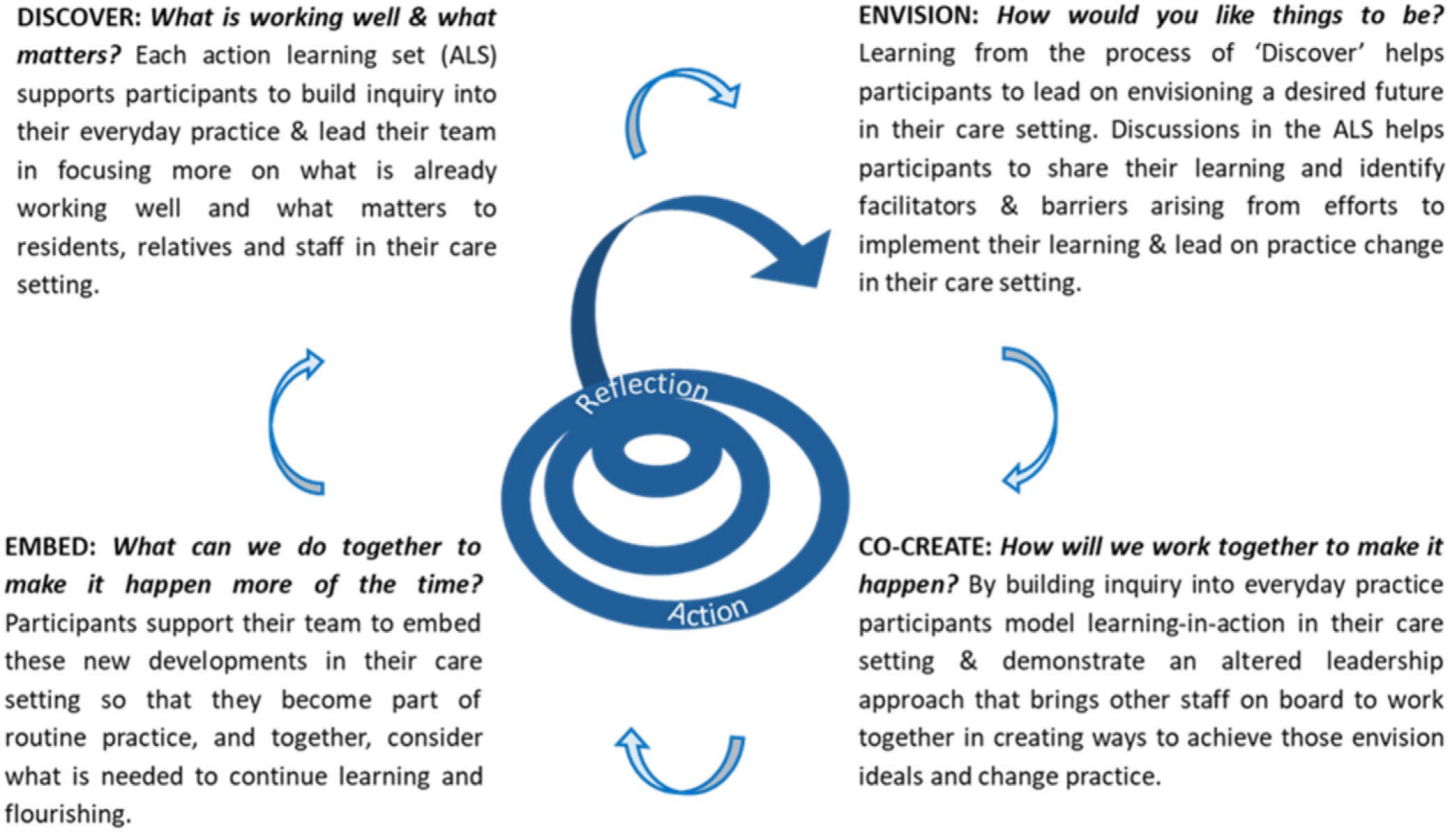
The methodology underpinning the delivery of the MHL Leadership Support Programme: Appreciative Action Research (AAR).

The methodology underpinning the MHL Leadership Support Programme is Appreciative Action Research (AAR, Dewar and Sharp 2013) and is embedded in the entire intervention. An AAR approach combines the key principles from both action research (Lewin 1946/1948; Reason and Bradbury 2009) and the four phases of Appreciative Inquiry: Discover, Envision, Co‐create and Embed (Dewar et al. 2017; Ludema et al. 2001) and promotes reflexive cycles, or iterations, between reflection and action. A diagrammatic representation of the MHL Leadership Support Programme's underpinning methodology is illustrated in Figure 1 below. Over the course of the programme, participants are introduced to a variety of MHL practice inquiry tools and approaches to support the four phases of AAR. They are encouraged to ‘give things a go’ and experiment with these approaches in their care setting to enact appreciative, relationship‐centred practice. This paper does not report on specific outcomes of the four phases of AAR but instead presents qualitative data collected at the end of the programme using two different sources: (1) one‐to‐one interviews and (2) facilitator field notes, to evaluate the impact of the MHL Leadership Support Programme on residents and families as perceived by programme participants.

#### 2.2.1 Practice Inquiry Tools and Resources Used to Deliver the MHL Programme

The variety of resources and approaches that participants are introduced during the workshops and action learning sessions form an integral part of the MHL Leadership Support Programme. These resources have been specially designed to spark self‐reflection, relationship building and engagement while also generating new and different insights into what works well and what can be done better. The tools support participants to bring caring conversations (described above in Table 1) to life, changing the nature of conversations with residents, relatives and staff. Participants are encouraged to use these tools and translate what they have learned during the workshops into practice in their own care settings. A snapshot of practice inquiry tools and resources used to deliver the MHL Programme is presented in Table 2.

**TABLE 2:** MHL practice inquiry tools and resources.

MHL practice inquiry tools and resources	Description
Emotional Touchpoints (Dewar et al. 2010)	Emotional Touchpoints are small cards with an emotion word written on each card for example, ‘trusted’ ‘upset’, ‘respected’, ‘frustrated’. They assist participants in exploring the experiences of residents, relatives and staff and support the person to tell their story and share their experience in a structured way.
Image cards/photo elicitation (Collier 1967, 1987)	A set of cards with generic images are used to open up conversations, support discussion, and facilitate enhanced articulation of meaning. Research has shown that the sharing generated through the use of images can be more detailed than that which occurs when only verbal means are used. The use of photo elicitation can help to build connections between people, as they share in real and meaningful ways, while staying safe and only sharing what they feel comfortable with.
‘I used to …. And now I ….’	During the course of the programme, facilitators support participants to reflect on changes they notice in themselves. Use of the sentence stem ‘I used to …. and now I ….’ provides the scaffolding that enables participants to identify examples of changes in their behaviour since starting the Programme and share their experiences and success stories.
Insights into Me	‘Insights into me’ is a resource designed to help staff initiate caring conversations with residents who have recently moved into the care home. It contains different sets of questions and prompts that have been created so that staff can find out what really matters to the resident and what makes any day a good day for them. It is designed to also support the resident in getting to know the staff members better. The resource contains a family tree so that the resident can populate the tree with the people they like having in their life.

### 2.3 Design

The study reported in this paper is an evaluation of the impact of the MHL Leadership Support Programme on people who live in care homes and their families as perceived by participants. A qualitative description study design was adopted (Sandelowski 2000) to provide straightforward descriptions of care home leaders' experiences of undertaking the MHL Leadership Support Programme. Qualitative description design was used as it places emphasis on the individual leader's lived experiences of implementing their learning from the programme and leading on practice change in their care setting. Qualitative post‐intervention data were collected at the end of the programme using one‐to‐one semi‐structured interviews with participants to evaluate the impact of the programme. The main aim was to gain an understanding of the impact of the programme on the participating leaders and the perceived impact that the programme had on residents and relatives. All 61 participants who completed the programme were invited to take part in the one‐to‐one interviews and of these (*n* = 56) participated. An interview schedule guided the discussions and explored the impact of the programme on participants' leadership skills and on their relationships with residents and families (see Appendix S2). All interviews were recorded and transcribed.

In addition to the one‐to‐one interviews, detailed field notes were captured by the MHL facilitators across all five cohorts of care home leaders during the course of the programme. The facilitator field notes were captured during each of the seven monthly action learning sessions and were written up in one final report when action learning had finished. The field notes provide structured and robust accounts, from participants, of practice improvements/developments in their care homes and feedback from staff, relatives and residents. Qualitative field notes enhance data and are considered a key element of rigorous qualitative research (Phillippi and Lauderdale 2018). The field notes were structured under several broad areas including introduction; background and local context; organisational factors; developing relationships; developing self; leadership, management and quality improvement outcomes; and conclusions. The field notes captured any particular observations, lessons learned and outcomes of actions taken forward by participants, along with their perceived impact on the well‐being of residents, staff and families. At the end of the programme, a validation day was held during which participants thematically analysed the data with the facilitator using the immersion–crystallisation process (Borkan 1999). This involved reading the data extracts, crystallising the key messages in data extracts, reflecting these back to each other and synthesising and corroborating the themes. Following this process, the facilitator of each cohort drafted a final report (*n* = 5). Participants in each of the five cohorts were invited by their facilitator to check the final report for accuracy and resonance with their experiences.

### 2.4 Sample

Participants were recruited from a region of the United Kingdom and consented to participate in the research study. The inclusion criteria were care home managers, deputy managers, rising stars and other staff with leadership roles in their care home settings. Eighty‐three participants were recruited to the study in October/November 2021. The study spanned a 10‐month period, and 61 participants completed it in July/August 2022. Data collection occurred between July and September 2022. Data were generated from care home leaders (*n* = 61), across five cohorts who completed the study. Table 3 below shows a breakdown of the number of participants in each of the five cohorts and their specific job roles.

**TABLE 3:** Number of participants in each cohort and their job roles.

Cohort	Care manager	Deputy manager	Nurse manager	Team leader/nurse/specialist nurse	Senior care assistant	Regional manager	Quality assurance manager	Participants per cohort
A	6		5					11
B	5	1						6
C	13		1	1				15
D	8	2		5	1			16
E	5	4		2		1	1	13
Total number of participants								61

### 2.5 Data Analysis

As presented above, this research study was informed by two discrete but interconnected strands of qualitative data, that is, data from individual interviews with participants, and data from facilitators' final reports. In order to analyse the data, a different approach was adopted for each data source and is described in the following sections of the paper.

#### 2.5.1 Individual Interviews

Thematic analysis was used to analyse the interview data. This involved putting the data through a set of processes consistent with Braun and Clarke's approach (2021) for identifying, analysing and reporting patterns or themes within data. Braun and Clarke's approach involves six key phases: 1. Familiarisation with the data; 2. generating initial codes; 3. generating initial themes from coded and collated data; 4. developing and reviewing themes; 5. refining, defining and renaming themes; and 6. producing the report. Interviews were transcribed verbatim, and the first author checked all transcripts against audio recordings to ensure accuracy. After reading and re‐reading transcripts for familiarisation, an initial coding framework was developed as a result of line‐by‐line coding and importing data into NVivo 12 qualitative data management software (QSR International 2018) by the first author. Two authors agreed on the final coding framework. Codes and themes were regularly discussed within the research team.

#### 2.5.2 Facilitators' Final Reports

**FIGURE 2:**
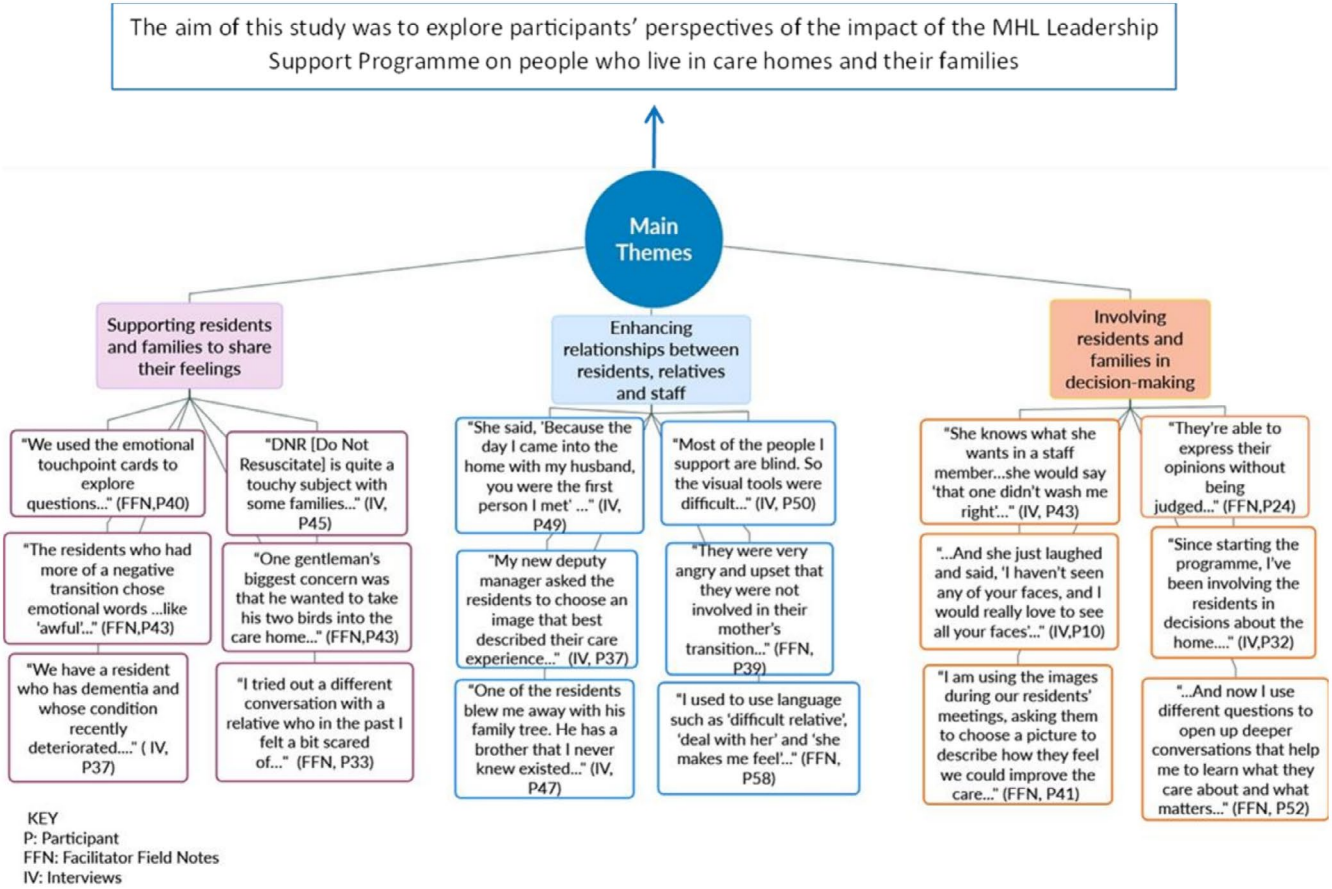
Cognitive map illustrating main themes mapped to the aim of the study.

Content analysis (Labuschagne 2003) was considered more appropriate to analyse the facilitators' final reports and the authors used a conventional content analysis approach (Hsieh and Shannon 2005). The rationale for using conventional content analysis was that data contained in the report documents had already gone through a process of co‐analysis by participants and facilitators who jointly analysed and interpreted the data, leading to a shared understanding. Although co‐analysis helped refine and validate initial interpretations of the data, conventional content analysis provided a structured way to systematically analyse and categorise the data, which helped in revealing additional patterns and insights that had not been identified during the co‐analysis process. While the interview transcripts and facilitator reports were analysed independently, the same issues were recurrent and the themes that emerged from the interview transcripts broadly aligned with the facilitators' final reports. Regular discussion within the research team helped to resolve any uncertainties or discrepancies. The themes generated served to integrate the data gathered by the two different methods of data collection. The results of the thematic and content analysis were brought together using the process of cognitive mapping. ‘A cognitive map is the representation of thinking about a problem that follows from the process of mapping’ (Eden 2004, 673). The cognitive map is presented in Figure 2 and illustrates how the themes with related extracts map back to the aim of the study.

### 2.6 Ensuring Rigour

The interviews were conducted by eight members of the MHL team. To maximise rigour and avoid bias, facilitators did not carry out interviews with participants from the cohorts they had been working with during the course of the programme. During the coding stage, two members of the research team viewed the original uncoded transcriptions and confirmed themes, thus ensuring that the interpretations represented the experiences of participants. Two researchers also independently reviewed the facilitators' final reports as part of the content analysis process. Following the selective coding process, and to maximise credibility, dependability and confirmability (Lincoln and Guba 1985), all members of the research team met to review the emerging themes and discuss alternative interpretations of the data. In order to enhance the trustworthiness of the data, any differences of opinion were discussed until consensus was reached.

## 3 Findings

The overall aim of this study was to explore participants' perspectives of the impact of the MHL Leadership Support Programme on people who live in care homes and their families. The programme appeared to have shone a light on transitions as a major life event for older people and their families. The thematic analysis of the interview data and the content analysis of the facilitators' final reports identified three key themes that revealed a chronology in relation to transitioning into a care home. The themes were (1) supporting residents and families to share their feelings; (2) enhancing relationships between residents, relatives and staff; and (3) involving residents and families in decision‐making. The following sections present the three key themes illustrated by selected excerpts from the interviews and the facilitators' final reports.

### 3.1 Theme 1: Supporting Residents and Families to Share Their Feelings

While the move to a care home was recognised by participants as a very difficult time for older people and their families, findings indicated that participation in the programme raised their awareness of the move as a major life event. There was also recognition that more could be done to support older people and their families during this challenging transition. Several participants explained how they were working with their staff to improve residents and families' experiences of transitioning to the care home. One participant explained how she used Appreciative Inquiry (described in Table 1, 6) to discover what was working well with transitions in their care home. Beginning with a positive story of when a transition worked well, she supported her team to envision how they would like to support future residents and families to settle into the care home. She co‐created a short questionnaire with her team to explore with four of her residents what their transitioning experience was like. The findings suggested that all four residents were kept outside the decision‐making loop in terms of their move to the care home. They had no knowledge of the care home environment, the bedrooms or what the social activities were. Based on these findings, the participant and her deputy prepared an information pack in advance of carrying out the next pre‐assessment in the hospital. She took photographs of the home and included these in the pack, along with the activity plan for residents. She also provided access to a Facebook page where the prospective resident and family could see what takes place in the home. At the pre‐assessment, they went through the information pack with the prospective resident and family and used ‘emotional touchpoints’ (described previously in Table 2) during their conversation:

We used the emotional touchpoint cards to explore questions like: How do you feel about coming into a nursing home? They picked the words ‘anxious’ and ‘worried’. We explored what their worries and concerns were. By the end of the meeting, the resident and their family felt reassured, and it eased their transition. (Participant 40)

Participants who previously would have had a hesitancy in opening up sensitive conversations with relatives and residents felt that the MHL practice inquiry tools such as the emotional touchpoints helped them to build their courage and confidence to initiate those conversations:

DNR [Do Not Resuscitate] is quite a touchy subject with some families and I have always been uncomfortable about bringing it up. Recently I asked a resident's relative to choose an emotion word that resonated with how they felt about DNR. They choose the word ‘scared’ and shared their thoughts and feelings about future decisions. That opened up a conversation and gave them the opportunity to tell me what they were scared about. Afterwards, they choose the words ‘relieved’ and ‘involved’ and said they had a better understanding. Maybe if they hadn't used the cards, they wouldn't have opened up to me the way they did, so it was it was very good. (Participant 45)

One participant described using **e**motional touchpoints and caring conversations (described in Table 1, 6) with four residents who had dementia. Three of the residents were emergency admissions which was quite stressful for both them and their families. Their pre‐assessments were all done over the telephone with a social worker and none of the families got to visit the home prior to the residents move:

The residents who had more of a negative transition chose emotional words and phrases on the cards, like ‘awful’ they were ‘angry’, ‘upset’, ‘scared’, ‘felt let down’ by their families. (Participant 43)

The participant stated that she took what she had learned from having caring conversations with the four residents and used it to enhance a new resident's transition to the care home:

One gentleman's biggest concern was that he wanted to take his two birds into the care home – this was identified in the pre‐assessment. Myself and the staff reassured him and facilitated his two birds being brought to the care home with him and he said ‘that's what's made it ‘home’ for him. (Participant 43)

A number of participants found that their staff were developing a greater awareness and insight into the huge impact moving into the care home was having on residents and their families. There was also evidence to suggest that the impact of transitioning from living in a residential care setting to moving into a nursing care setting was viewed by participants as equally challenging for the resident and their family:

We have a resident who has dementia and whose condition recently deteriorated. She needed to be moved from the residential area of the care home, where has she has been living since her admission, to the nursing area of the care home. The resident's daughter was very upset about this move, so I met with her to explore how she was feeling using the caring conversations. We were both able to express how we felt about the move and gain an understanding about how it would best meet her mum's needs by providing the specialised care she required. This really helped to facilitate a smooth transition for both the resident and her daughter. (Participant 37)

The findings indicated that participants had become more confident and courageous in modelling appreciativeness, sharing their feelings and asking others how they feel:

I tried out a different conversation with a relative who in the past I felt a bit scared of… I asked her how she was and shared a bit about myself and that I felt a bit scared speaking to her. She was surprised at this. I learned what was going on for her and the different pressures she was having. From this different conversation our relationship has really improved. Staff have seen my approach and it has helped them to engage with her too. (Participant 33)

During the course of the programme, facilitators supported participants to reflect on changes they were noticing in themselves using the sentence stem ‘I used to…and now I…’ (described in Table 2, 7). The findings indicated that by using the ‘I used to and now I' approach, participants were able to reflect on the reciprocity of sharing feelings with others:

I used to hide how I was feeling as I thought it was showing vulnerability and now I feel comfortable sharing my feelings and it helps others to do the same. (Participant 49)

I used to close down and avoid relationships with relatives who I felt a bit nervous to engage with, now I try to open up conversations with them by sharing how I feel and asking them more about what matters to them. (Participant 58)

I used to ask the person who made a complaint how they felt about this… I still do but now I share how I felt about receiving this. (Participant 33)

The ‘I used to and now I' approach was also a very powerful way of helping participants to appreciate how much their own practice had changed over the course of the programme and the positive impact of this change on residents and relatives.

### 3.2 Theme 2: Enhancing Relationships Between Residents, Relatives and Staff

It was evident from the findings that getting to know relatives and building meaningful connections began during the resident's transition to the care home. Participants who used caring conversations during the initial assessment explained that this approach really helped residents and their families to feel supported and much more at ease about the move into the care home. One participant described how using this approach had enhanced her relationship with the wife of a resident who had been admitted from his home. The participant explained that when the resident's wife came to visit her husband, she just ‘popped’ into the office to see the participant (manager), sat down and had a friendly chat. The participant said that she then decided to do some discovery work to find out what had helped the resident's wife feel so comfortable and at home:

She [*resident's wife*] said, ‘Because the day I came into the home with my husband, you were the first person I met …you welcomed me and involved me’. She puts it down to being involved in that whole transition from home into the care home, of being connected…of getting to know her as well as her husband and that she always feels safe when she comes into the care home This is the relationship we are aiming for with the family of every resident. (Participant 49)

Another participant shared a story about a resident who had recently moved into the care home. The resident's transition was very traumatic for her and was done without her knowledge or consent. She had three daughters but had stopped communicating with two of her daughters prior to her admission to the care home. There was only one daughter involved in her care and the other two estranged daughters felt very upset and were unaware of their mother's transfer. The resident was very unsettled and anxious following her transfer, and after a short period of time, she requested that the staff contact her other two daughters to help her settle in. The participant explained that she and her colleague met with both daughters:

They were very angry and upset that they were not involved in their mother's transition. It was a very difficult conversation and we did all we could and listened to them… I hadn't started the programme at that stage and felt I didn't have the tools to manage the conversation and the process with the daughters. (Participant 39)

The participant went on to explain that, during her time on the MHL Programme, she arranged to meet with both daughters again. She described ‘having the courage*’* during the meeting, to explain to them that she was currently undertaking the programme and she guided the conversation using an appreciative approach. She used ‘Image Cards’, (described previously in Table 2, 7) to explore with both daughters how they had felt at the time of their mother's move from the hospital to the care home:

One daughter chose the image of a monkey in a cage to show how she felt, and the other daughter picked the image of a lock and key. We used the emotion cards to explore how they felt we could have improved the transition process at the time. Both daughters said that if we had used this approach, it would have eased their minds and they'd have felt more involved. This feedback is helping us to improve transitions for future residents and relatives. (Participant 39)

Several participants described how they modelled this approach with their staff, empowering them to engage in more caring conversations with residents and explore feelings about their care experience:

My new deputy manager asked the residents to choose an image that best described their care experience. One resident chose an image with people sitting on a park bench and another chose the card with a number of people smiling and jumping into the air. The residents really thought about the picture they were choosing, and they talked so much about what it meant for them. The conversations were richer than before. The deputy continues to use this approach in resident meetings and has coached other staff in using these tools. (Participant 37)

Evidence in the findings also indicated that participants modelled an appreciative approach with their activity therapists, using the Image Cards to elicit residents' views on activities that provided meaning for them. However, a few participants highlighted that this is not an appropriate approach for residents who have a visual impairment or are blind:

Most of the people I support are blind. So, the visual tools were difficult, you know. Because you're relying on sight, so I think that was a bit of a barrier… (Participant 50)

Since photo elicitation is dependent on the person having sight, participants said they found it difficult to use the Image Cards with all their residents to fully connect and find out what mattered to them;

I'm taking a bit of time to do a work around. I'm using the emotion words on the cards to say the words out so they know what the choice is and give them time to choose…this is encouraging more connection and emotional discussion with my service users for their own well‐being…this change is coming from the impact of me doing the programme. (Participant 59)

Acknowledging the person's emotions was viewed by this participant as being fundamental to enhancing connections with the residents. While the visual tools were difficult for residents to use, the participant was able to adapt what she learned from being on the programme and use the emotion words to explore with residents what mattered most.

The findings indicated that, by being appreciative and initiating caring conversations with residents, participants were able to enhance connections and, in some cases, bring forward new information that had not been revealed in the past. A participant described how she had been working with most of the residents in her care home for 25 years. She explained that her residents were adults with a learning disability. She was introduced to the MHL ‘Insights into Me’ resource (described in Table 2, 7) when she started the programme and decided to use it as it allows for a deeper delve into every aspect of the resident's day and provides invaluable information about what really matters to the person. The ‘Insights into Me’ resource contains a family tree so that the resident can populate the tree with the people they like or would like to have in their life. The participant went on to explain that she showed the visual representation of the family tree to one resident who rarely speaks, and when he saw it, he called out a man's name:

One of the residents blew me away with his family tree. He has a brother that I never knew existed, until he did the family tree in the ‘Insights into Me’. This resident is here twelve years. He came from school as soon as he turned nineteen. I have never met his brother and he never comes to visit…This document gets down to the essence of the person and that's what My Home Life is all about. We're now rolling out this document to all residents and their key workers. (Participant 47)

The participant explained that after a considerable length of time she managed to get in touch with the resident's brother. During the course of the interview, the participant shared that the resident's brother had been to visit on a few occasions.

There was also evidence in the findings that the programme had raised participants' awareness of how the language they use can change the nature of the conversations they have with staff, particularly when speaking about relatives and residents. By using the ‘I used to … and now I …’ approach, participants were able to identify examples of changes in their language since starting the programme:

I used to use language such as ‘difficult relative’, ‘deal with her’ and ‘she makes me feel’, when talking about going into a meeting with a relative and now I use words such as ‘speak with her’, ‘I feel’ and ‘a relative that I find it a little difficult to speak with. (Participant 58)

I used to say to staff my pet hate was when they used language such as dear or love when talking to residents – I still feel uncomfortable when I hear this, but now I try to discuss this with staff rather than telling them it's wrong. (Participant 28)

Participants felt they were much more attuned to the use of language in their care settings and this included their own language and that used by others. There was evidence to show that they thought more about how things were said and how they might be heard by others, and how language can shape the culture in their care home.

### 3.3 Theme 3: Involving Residents and Families in Decision‐Making

Data from the interviews revealed that the programme helped participants to realise that, by focusing attention on relationships and enhancing connections, the residents, relatives and staff started to appreciate their own part in the bigger picture of life in the care home. This began to generate ideas about what might be possible in the home to enhance quality of life. One participant who had recently taken up a leadership post in her care home was recruiting new staff. She explained how she had adopted an appreciative approach to involve residents in the recruitment process. She spoke about one of her residents who she felt had the required strengths that would help make this happen. The resident agreed to sit on the interview panel and ‘run her eye over*’* the candidates. The participant explained that this resident would often comment on the different skills of various staff members and sometimes she would highlight an individual who she felt required more training in a specific aspect of care delivery:

She knows what she wants in a staff member…she would say ‘that one didn't wash me right…she needs more training’…and then she'll send me off to chastise the person because they didn't do it right…Placing the resident at the centre of the service is very much how we'll focus and move forward. (Participant 43)

The ethos of the programme is one of involving residents and families in an inclusive process of shared decision‐making, not only about treatments and care but also about all aspects of life in the care home:

Since starting the programme, I've been involving the residents in decisions about the home. We asked them, what does home mean for you? What does home look like? What does home feel like? And a lot of them actually wanted a stove and they wanted an actual fire that they can sit at. I've taken their requests to senior management to try and see how we can safely create what they want…We're also involving the residents in a competition about renaming two wings in the home and some of the names they've come up with are local names and that's great! (Participant 32)

Many of the interviews with participants took place during the Spring/Summer of 2022 and masks were still being worn by the staff, families and visitors in care homes to minimise the effects of the COVID‐19 pandemic. One participant described how she used appreciative inquiry in the residents' meeting to discover what would enhance their quality of life in the home. The residents unanimously requested that the staff stop wearing masks as they really wanted to see their faces again. The participant then undertook further discovery work to find out how the relatives and staff felt about this. Everyone confirmed that they wanted the wearing of masks stopped with the understanding that ongoing risk assessments would be carried out. One relative, however, felt she could not consent to the cessation of wearing masks. The relative's sister was a resident in the home: she was 60 years of age, was extremely ill and receiving palliative care. The relative requested that the manager (participant) go with her and ask her sister how she felt. Together, they explained to the resident what had been discussed in relation to the wearing of masks. The participant reassured the resident that the staff would continue to wear masks when caring for her:

…And she just laughed and said, ‘I haven't seen any of your faces, and I would really love to see all your faces. I don't want to die seeing masks!’…We thought we were doing a good thing protecting her, but she was going to die seeing masks. She died, but she got to see our faces. (Participant 10)

By keeping the resident at the centre of decision‐making, and based on resident preference, the staff stopped wearing masks.

It was evident in the findings that establishing trust between residents, families and staff was a central component of shared decision‐making in the care homes. A participant explained how she placed a small artificial tree at the front door of her care home and invited relatives to write comments/suggestions on leaf‐shaped labels about various aspects of life in the care home, and hang them on the tree:

They're able to express their opinions without being judged and we don't have to know who wrote it. So that's actually quite a big difference for relatives to have that opportunity to put any comments and suggestions down. And you know, reading all the comments helps to improve staff morale. You don't realise that, gosh this is how they feel, and it's really nice to know. (Participant 24)

Participants found that, by using particular inquiry tools such as the image cards, they asked fewer questions than previously, and listened more deeply which helped to build trust and enhance relationships:

I am using the images during our residents' meetings, asking them to choose a picture that would describe how they feel we could improve the care. And that's amazing… it's opening up a lot of valuable information about what they want. Whereas before I would ask the question ‘Do you feel that the care is dignified and respectful?’ and they would say ‘yes’ or ‘no’. Now, they're feeling more involved…they're getting a bit more excited about it. (Participant 41)

Evidence in the findings demonstrated that participants were developing new skills in seeking out more in‐depth knowledge about the person and what truly matters to them. Some of these improved outcomes were described by participants using the ‘I used to…and now I…’approach.

I used to ask clients who were moving on from our service what they needed and wanted and where they wanted to live and now I use different questions to open up deeper conversations that help me to learn what they care about and what matters, such as what kind of community would you value, what helps you to feel other people are fond of you – this question connects emotionally and is something I would not usually ask. (Participant 52)

By focusing attention on relationships in their everyday work, participants reported that they had developed greater confidence in engaging with residents and families and seeking out their views about enhancing quality of life in the care home.

## 4 Discussion

This study set out to explore the impact of the MHL Programme on care home residents and relatives from the perspectives of the participating leaders. One of the key messages that have emerged from the study is the power of the programme to heighten awareness about the importance of the relational aspects of practice in the care setting. The findings reveal that the MHL tools and approaches used to facilitate appreciative caring conversations, enabled participants to develop an attitude of inquiry, explore the hopes, values and feelings of residents and relatives and support residents and relatives in voicing their perspectives about what matters most to them.

### 4.1 Modelling the Role

It was evident from the study that participants were developing a greater insight into their own behaviours and relational skills. This was demonstrated in how they supported, modelled and encouraged staff to use the MHL tools and approaches to enact appreciative relationship‐centred care. Modelling the role has been described as ‘*facilitator of inquiry process’* (Sharp et al. 2018, 234). Modelling the role was evident in how participants displayed the courage and confidence to share their own vulnerability in conversations with relatives and residents which helped to build trust and enhance relationships. Evidence from a recent literature review on shared decision‐making in care homes highlighted the importance of trust as central to collaborative relationships and family members' ability to participate in shared decision‐making (Lynch et al. 2022).

The concept of facilitation has been viewed in the literature as *‘a process of providing support to individuals or groups to achieve beneficial change’* (Petrova et al. 2010, 38). A key component of providing support to enable beneficial change to occur is providing a ‘sense of security’ (Nolan 2006). This was demonstrated in how participants facilitated caring conversations and supported residents and relatives to feel safe in expressing the emotions they were feeling about the changes to their lives after the move to a care home. It can be difficult for people to find the language and share the complex range of emotions they are feeling without a prompt to facilitate articulation of meaning. Participants highlighted the value of the MHL tools, for example, the image cards and the emotional touchpoints, in enabling residents and relatives to reveal their inner thoughts and feelings about the move, especially during the initial stages of their transition.

Findings in the literature around transitions show that a person's transition to the care home can be negatively impacted if there is a high turnover of staff (Krein et al. 2022). Recent research highlights the need for staff consistency and recommends that a key member of staff facilitates the transition during that critical period when the person first moves into the care home (O'Neill et al. 2020a). A supportive leadership style by care home leaders is identified as a significant predictor that can positively impact staff turnover (Backman et al. 2021; Eltaybani et al. 2018).

### 4.2 Relationship‐Centred Care

Research evidence suggests that education for leaders tends to focus on legislative/organisational needs rather than supporting them to develop the skills they need to negotiate the complex, and often conflicting, emotional and relational issues of their work (Kelly and Kennedy 2017). While a care home is a place people often call home and where people are supported in having a home‐like experience, it is also a clinical environment where professional healthcare is monitored, and standards have to be adhered to. It is important, therefore, that the models of leadership that have been developed for acute care and management situations are not directly imposed upon the care home sector (Lynch et al. 2018). Such models do not fit with the philosophy of care in care homes which is based on a shared set of concepts that are relevant and meaningful to disparate groups of people both giving and receiving care (i.e., residents, relatives and staff). These concepts are captured in the Senses Framework (Nolan 2006) which suggests that we need to consider what gives each individual involved in the care experience a sense of security, belonging, continuity, purpose, achievement and significance. Consequently, there is a need for a model of leadership that holds these principles and works with the knowledge base that exists around relationship‐centred care (Bourgeault et al. 2022; Zonneveld et al. 2021). The MHL Programme was specifically designed for care home managers, deputy managers, ‘rising stars’ and other staff with leadership roles in their care homes. The findings have significant applicability internationally across all long‐term care settings where staff work collaboratively with service users and families to create a relationship‐centred culture. While the views of participants are invaluable in providing rich data about the impact of the programme, part of our ongoing inquiry will include the views and experiences of residents, relatives and staff and in doing so, provide a triangulation of impact evidence. Further research could strengthen the generalisability of the MHL Programme.

## 5 Limitations

The delivery of the programme was directly impacted by staffing crises during and following the spread of the Omicron variant of COVID‐19. This impacted attendance, and for several participants, it also had an impact on their ability to complete the programme. The results of the study are based on the participants' self‐reported or perceived impact of the programme. However, further studies to directly explore the views and experiences of care home residents and relatives on the impact of interventions such as leadership support programmes are recommended.

## 6 Conclusion

The care home, as a community for the people who live there, their families who visit and the people who work there, embodies a complex array of relationships, interactions and connections. Leadership in this environment is complex, multidimensional and influenced by multiple internal and external factors. Supporting care home leaders to develop leadership capability within this context requires a movement towards leadership models that are better suited to the principle of relationship‐centred care and the concept of appreciation and collaboration. The findings presented in this paper provide significant evidence of the impact of the MHL Leadership Programme on care home leaders' ability to enhance the care experience of residents and relatives and create a relationship‐centred culture.

## Author Contributions

S.M'I. and A.R. contributed to study design; P.S. contributed to theory development; B.L., S.P., R.B., M.O'N., C.M'C., E.‐R.B., A.C., U.H. and D.M. contributed to data collection; B.L. and A.R. conducted formal analysis; B.L. drafted the manuscript; A.R. and S.P. critically revised the manuscript for important intellectual content; all authors read and approved the final manuscript.

## Ethics Statement

Ethical approval for this research study was received from the University Filter Committee (Reference FCNUR‐21‐039). Participation in the study was voluntary and informed consent procedures were designed to provide the participants with sufficient information so that they could make an informed decision about the potential inconveniences and benefits of participating in the study. Participants were assured of anonymity, confidentiality and their right to withdraw.

## Conflicts of Interest

The authors declare no conflicts of interest.

## Supporting information


**Appendix S1:** opn70054‐sup‐0001‐AppS1.docx.

**Appendix S2:** opn70054‐sup‐0002‐AppS2.docx.

## Data Availability

Anonymous versions of the data sets used are available from the corresponding author on reasonable request.
