# Supplementary material for: The Impact of a Leadership Support Programme on Care Home Residents and Their Families: A Qualitative Study From the Perspective of Participating Care Home Leaders
Source: Int J Older People Nurs. 2025 Nov 16;20(6):e70054. doi: 10.1111/opn.70054 (PMC12620537; doi:10.1111/opn.70054)
Supplement: Supplementary file 1 — Appendix S1: opn70054‐sup‐0001‐AppS1.docx. [file OPN-20-e70054-s001.docx]

| **Day 1 Workshop** | **Day 2 Workshop** |
| --- | --- |
| - Broad aims of My Home Life (MHL) initiative - Outline of specific aims and values of the Leadership Support Programme - Introduction to the evidence base - Connections to research outputs – general overview of how we gather data - Your Quality of Life - Coping with Stress - Focusing on relationships | - Exploring relationships within the care home and wider stakeholders - Introduction to the seven Cs:   Becoming **C**ourageous  **C**onnecting emotionally  Becoming **C**urious  **C**ollaborating  **C**onsidering other perspectives  **C**ompromising  **C**elebrating |
| 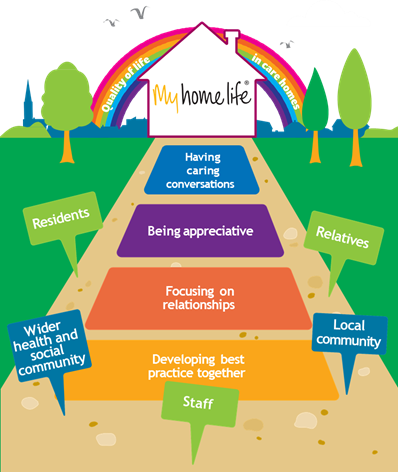 | |
| **Day 3 Workshop** | **Day 4 Workshop** |
| - Review of Day 1 & Day 2 - Development and change in practice using the seven C’s. - Facilitating quality improvement: - Introduction to Appreciative Inquiry: Discover; Envision; Co-create; Embed - Introduction to the value of the MHL Best Practice Themes to narrow the focus of the inquiry. - Turning theory into action: what works well and what it could look like at its best. - Using MHL practice inquiry tools | - Connecting emotionally using MHL practice inquiry tools - Examining how the improvement process can both embed and model an ethos of relationship-centred care and help take forward change. - Based on what the participants had collectively identified on day 3, participants consider one thing that could help them move towards their vision. - Bringing everything together: summarising the narrative within the four days. - Introduction to Action Learning. - Introduction to Quality Improvement (QI) & planning of QI Initiatives going forward. |
| **Please note:** The day of delivery for topics may move within the 4 day workshops and this flexibility will be led by the requirements of each cohort of participants. | |

**Appendix S1:**  **An overview of the content of the 4 day workshops**
