# Supplementary material for: The Impact of a Leadership Support Programme on Care Home Residents and Their Families: A Qualitative Study From the Perspective of Participating Care Home Leaders
Source: Int J Older People Nurs. 2025 Nov 16;20(6):e70054. doi: 10.1111/opn.70054 (PMC12620537; doi:10.1111/opn.70054)
Supplement: Supplementary file 2 — Appendix S2: opn70054‐sup‐0002‐AppS2.docx. [file OPN-20-e70054-s002.docx]

**Appendix S2: MHL Leadership Support Programme/Interview guide**

**Part A: Questions about the MHL Leadership Support programme.**

1. How would you describe your overall experience of the My Home Life Leadership Support Programme?
2. What aspects of the programme would you celebrate?
3. What aspects of the programme do you feel could be done differently?
4. How do you think the programme impacted on you as a leader/manager?
5. Have you observed any changes in your staff?
6. How do you feel it has it impacted on your residents?
7. How do you feel it has it impacted on your relatives?
8. Are you currently working on any other quality improvement initiatives outside of the cohort topic within your care home?
9. How has the MHL Leadership Support programme helped you with this?
10. Would you recommend the MHL Leadership Support programme to another colleague?
11. What do you feel can be done to improve communication between care homes and the wider health and social care community?
12. What do you think needs to be done to promote more movement of residents out into the community and the community into the care home?

**Part B: General Questions**

1. What has it been like to be in your role over the past year?
2. What do you think has changed / will change as a result of the Covid 19 pandemic?
3. What would you say are the top main things that are working well for you in your role as a care home manager?
4. What would you say are the main things you would like to change/notch up in your role as a care home manager?
5. In your experience of the RQIA inspection process what do you feel works well?
6. What would you like to see changed?
7. What would help support more shared decision-making in your care home?
8. Would you like to make any further comments?

***Thank you.***
